# Detection of asymptomatic *Leishmania* infection in Bangladesh by antibody and antigen diagnostic tools shows an association with post–kala-azar dermal leishmaniasis (PKDL) patients

**DOI:** 10.1186/s13071-021-04622-8

**Published:** 2021-02-17

**Authors:** Sophie I. Owen, Faria Hossain, Prakash Ghosh, Rajashree Chowdhury, Md. Sakhawat Hossain, Chris Jewell, Isra Cruz, Albert Picado, Dinesh Mondal, Emily R. Adams

**Affiliations:** 1 Department of Tropical Disease Biology, Liverpool School of Tropical Medicine (LSTM), Liverpool, UK; 2 Nutrition and Clinical Services Division, International Centre for Diarrhoeal Diseases Research (icddr,b), Dhaka, Bangladesh; 3 Lancaster Medical School, Lancaster University, Lancaster, UK; 4 Foundation for Innovative New Diagnostics (FIND), Geneva, Switzerland; 5 Present Address: National School of Public Health, Instituto de Salud Carlos III, Madrid, Spain

**Keywords:** Visceral leishmaniasis, Elimination, Asymptomatic *Leishmania* infection, Diagnostics, *Leishmania* antigen ELISA, qPCR

## Abstract

**Background:**

Asymptomatic *Leishmania* infections outnumber clinical infections on the Indian subcontinent (ISC), where disease reservoirs are anthroponotic. Diagnostics which detect active asymptomatic infection, which are suitable for monitoring and surveillance, may be of benefit to the visceral leishmaniasis (VL) elimination campaign on the ISC.

**Methods:**

Quantitative polymerase chain reaction (qPCR), loop-mediated isothermal amplification (LAMP), and the direct agglutination test (DAT) were carried out on blood samples, and the *Leishmania* antigen ELISA was carried out on urine samples collected from 720 household and neighbouring contacts of 276 VL and post–kala-azar dermal leishmaniasis (PKDL) index cases, with no symptoms or history of VL or PKDL, in endemic regions of Bangladesh between September 2016 and March 2018.

**Results:**

Of the 720 contacts of index cases, asymptomatic infection was detected in 69 (9.6%) participants by a combination of qPCR (1.0%), LAMP (2.1%), DAT (3.9%), and *Leishmania* antigen ELISA (3.3%). Only one (0.1%) participant was detected positive by all four diagnostic tests. Poor agreement between tests was calculated using Cohen’s kappa (*κ*) statistics; however, the *Leishmania* antigen ELISA and DAT in combination captured all participants as positive by more than one test. We find evidence for a moderately strong association between the index case being a PKDL case (OR 1.94, *p* = 0.009), specifically macular PKDL (OR 2.12, *p* = 0.004), and being positive for at least one of the four tests.

**Conclusions:**

*Leishmania* antigen ELISA on urine detects active asymptomatic infection, requires a non-invasive sample, and therefore may be of benefit for monitoring transmission and surveillance in an elimination setting in combination with serology. Development of an antigen detection test in a rapid diagnostic test (RDT) format would be of benefit to the elimination campaign.
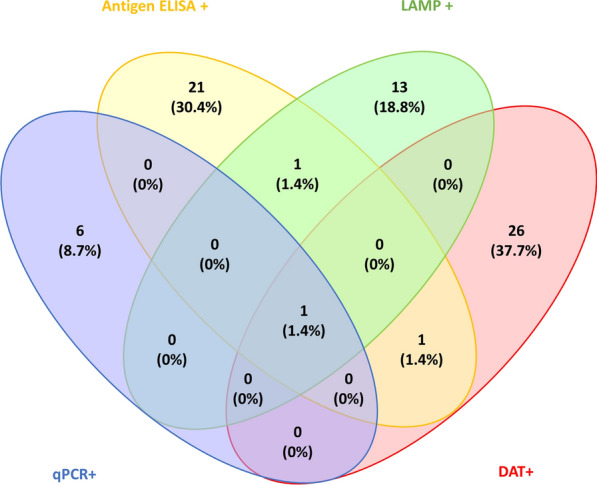

## Background

Infection with the parasite *Leishmania donovani* (*L. donovani*) usually manifests as asymptomatic infection with a small risk of progression to visceral leishmaniasis (VL), which in the absence of treatment is considered fatal [1]. Progression from asymptomatic infection to symptomatic disease was estimated to be between 5.6 and 15.2% in individuals with high anti-*Leishmania* antibody titres, as measured by the direct agglutination test (DAT), in India and Nepal [2]. Globally, the ratio of asymptomatic to symptomatic VL varies [3]. In Bangladesh, the number of asymptomatic cases was found to outnumber symptomatic cases by 4 to 1 [4].

Asymptomatic infection is of importance to VL endemic regions of the Indian subcontinent (ISC—India, Nepal, and Bangladesh), where the disease has been the target of an elimination campaign since 2005 [5, 6]. The epidemiology of VL is cyclical, and outbreaks occur approximately every 15 years on the ISC [7]. Asymptomatic carriers may represent a potential source of transmission in a region where parasite reservoirs are anthroponotic [8]. However, it is yet to be determined whether asymptomatically infected humans are infective to sand flies. A study in a small number of asymptomatically infected dogs showed that *L. infantum* parasites were transmittable to sand flies [9]; however, no human data with *L. donovani* have yet been recorded. Sixteen (8.2%) asymptomatic individuals who converted to VL within 2 years in a study in Bangladesh were found to have significantly higher anti-rK39 antibody titres than their counterparts who did not progress [10].

The rK39 enzyme-linked immunosorbent assay (ELISA), rK39 rapid diagnostic test (RDT), and the DAT measure the presence of anti-*Leishmania* antibodies [11–14]. These antibodies have been found to persist for months or years after infection, with patients in the VL endemic region of Muzaffarpur, India found positive by rK39 RDT (39.0%) and DAT (53.0%)  ≥ 15 years post-treatment [15]. Therefore, a clinical history is required to determine whether a positive result is due to active or previous infection, or a previous asymptomatic infection that will not progress to disease. Tests which detect active infection, such as quantitative real-time polymerase chain reaction (qPCR), loop-mediated isothermal amplification (LAMP), or *Leishmania* antigen ELISA, could be used as tools to monitor active asymptomatic infection and quickly identify areas with increasing active transmission.

Highly sensitive qPCR was shown to be an effective technique for diagnosis of VL and monitoring of treatment response, and could be of value in an elimination setting [16]. LAMP enables the robust, fast, simple, and highly specific amplification of nucleic acids and does not require a thermocycler or cold chain; the Loopamp™ *Leishmania* Detection Kit (Eiken Chemical Co., Japan) targets both the 18S rDNA and kinetoplast DNA (kDNA), and was previously demonstrated to have sensitivity of 92% in patients with suspected VL in Ethiopia [17]. Similarly high sensitivity of 98% and 100% was seen in a study in Sudan with the Loopamp™ *Leishmania* Detection Kit when DNA was extracted from peripheral blood using boil-and-spin and QIAamp DNA mini kits (Qiagen, Hilden, Germany), respectively [18]. Finally, the *Leishmania* antigen ELISA (Clin-Tech, Guilford, UK) detects low-molecular-weight *Leishmania* carbohydrates excreted in the urine and therefore detects active infection and uses a non-invasive sample type. A study found sensitivity to range from 77% (*n* = 13) in Bangladesh to 87% (*n* = 46) in Ethiopia, although more data are needed to evaluate this assay [19]. The clinical utility of the *Leishmania* antigen ELISA is yet to be determined in an asymptomatic population.

To determine the utility of the DAT, kDNA qPCR, Eiken LAMP, and the *Leishmania* antigen ELISA for monitoring and surveillance of asymptomatic *Leishmania* infection in an elimination setting, we tested samples collected from household or neighbouring contacts of index cases from endemic regions of Bangladesh. In this study, asymptomatic infection is defined as being positive for at least one of the aforementioned tests. Risk factors for asymptomatic infection were also investigated. We then compare the measure of prevalence with that obtained from a latent class analysis, in which the test characteristics of our four tests are formally synthesised through the use of a probability model.

## Methods

### Asymptomatic visceral leishmaniasis clinical samples

Blood and urine samples from 720 clinically healthy household and neighbouring contacts in adjacent households of 276 VL or PKDL index cases (between 1 and 8 contacts per index case), aged 5 to 60 years, with no symptoms or history of VL or PKDL, were collected between September 2016 and March 2018. Symptoms considered as exclusion criteria included fever, presence of a rash, enlargement of the spleen or liver, and weakness, among others. The study was conducted in the VL endemic districts of Mymensingh, Gazipur, Tangail, Narail, Jamalpur, Pabna, and Brahmanbaria in Bangladesh. Symptoms considered included presence of fever, rash, loss of appetite, weight loss, lymph node enlargement, and abdominal enlargement and pain. Demographic and clinical parameters were recorded. Blood and urine samples were transported to Dhaka using a cold chain for processing and laboratory analysis using DAT and LAMP. Urine and DNA samples for *Leishmania* antigen ELISA and qPCR, respectively, were transported on ice to the UK from Bangladesh and stored at −20 °C until testing.

### DNA extraction

DNA was extracted in three different ways: (1) DNA was extracted from 100 µl whole blood and eluted in 200 µl buffer using DNeasy blood and tissue DNA extraction kits (Qiagen, Hilden, Germany) as per the manufacturer’s instructions. (2) Boil-and-spin extractions were carried out by pretreating whole blood samples with sodium dodecyl sulphate (SDS). Briefly, 10% SDS solution was mixed with blood to a final concentration of 5% and stored at −20 °C. Once defrosted, samples were inverted 10 times and allowed to stand at room temperature for 10 min. Samples were further inverted, and 400 µl of distilled water was added before incubation at 90 °C for 10 min. Tubes were then centrifuged at maximum speed for 3 min, and the supernatants stored for testing. (3) DNA was extracted from dried blood spots (DBS). Whole blood was air-dried onto Whatman filter paper (GE Healthcare Life Sciences, Buckinghamshire, UK) for 30 min at room temperature and stored in individual bags. Discs of 7 mm were punched out of the paper and added to an Eppendorf tube with 50 µl of double-distilled water. Tubes were incubated at 90 °C for 10 min followed by centrifugation for 3 min at maximum speed. Supernatants were stored at −20 °C for testing.

### qPCR

Real-time PCR (qPCR) was performed on DNA extracted from whole blood using Qiagen DNeasy kits (Qiagen, Germany) [17]. An aliquot of 1.25 μl DNA was added to 11.25 μl of an amplification mixture containing 2.5 μl QuantiFast master mix (Qiagen, Germany), 0.4 μM kDNA forward primer, 0.4 μM kDNA reverse primer, and 0.2 μM kDNA FAM probe. Amplification was performed on a Qiagen Rotor-Gene Q system with the following reaction conditions: 5 min at 95 °C, followed by 40 cycles of 15 s at 95 °C and 30 s at 60 °C. Data were analysed using the Rotor-Gene Q series software (Qiagen, Germany). Standard curve analysis was performed using *Leishmania donovani* DNA (positive control), and the data were used to set a qPCR threshold. Samples with cycle threshold (Ct) < 34 were considered positive to reduce detection of non-specific amplification.

### Loop-mediated isothermal amplification (LAMP)

LAMP was run on DNA extracted from whole blood using the DNeasy blood and tissue DNA extraction kits (Qiagen, Germany), boil-and-spin extraction, and extraction from dried blood spots as described above. Loopamp™ *Leishmania* detection kits (Eiken Chemical Co., Ltd., Tokyo, Japan) were used. Samples to be tested were made up to total volume of 30 µl by adding 3 µl DNA sample to 27 µl of water. The lids of the tubes were then closed, and the sample were mixed with the master mix contained in the tube cap by inverting the tubes and leaving them to stand for 2 min cap-side down. The tubes were inverted five times, spun down, and incubated at 65 °C for 40 min, then 80 °C for 5 min. Results were visualised under blue LED light illumination, using the fluorescence visual check unit of the HumaLoop M incubator (HUMAN, Wiesbaden, Germany). Results were read by two technicians blinded to each other. A third technician was consulted in the event of disagreement, and the majority decision used.

### Direct agglutination test (DAT)

The DAT was carried out in Bangladesh and performed as previously described [20]. Following a dilution of sera 1:100, the samples were further diluted in eight twofold serial dilutions. Where samples did not react in the first dilution, the end titre was read as < 1:200. Where samples still reacted at the final dilution, the end titre was read as > 1:25,600. The threshold for a positive DAT result was set at ≥ 1:1600 as previously used by Hasker et al. for detection of asymptomatic infection [20].

### *Leishmania* antigen ELISA

The *Leishmania* antigen ELISA (Clin-Tech, Guildford, UK) was performed on urine samples as per the manufacturer’s instruction. Briefly, samples were diluted 1:20 with assay diluent. One hundred microlitres of antigen calibrators and diluted samples was added to a pre-coated 96-well plate and incubated at 37 °C for 30 min. Following four washes, 100 µl of working strength tracer was added to the wells and incubated at 37 °C for 30 min. Following a further four washes, 100 µl of TMB substrate was added to each well and incubated uncovered between 18° and 25 °C for 30 min. One hundred microlitres of stop solution was then added to each well. A standard curve was included on each plate. The optical densities (OD) were read at 450 nm and blanked on air or with the 620 nm reading within 30 min of addition of stop solution. Four-parameter curve-fitting software was used to calculate the concentration (UAU/ml) of each sample. IBM SPSS Statistics version 24 software was used to generate receiver-operating characteristic (ROC) curves using 720 asymptomatic cases and 80 VL cases to determine the threshold in UAU/ml that gave a sensitivity of 98.8% and a specificity of 96.7%. The area under the curve (AUC) was calculated.

### Statistical analysis

Data were analysed in R Studio version 1.1.456. Discrete variables were summarised as counts and percentages. Continuous variables were summarised as the median and interquartile range (IQR). The software package ‘Venny’ was used to create Venn diagrams for comparison of diagnostic tests [21].

Percentage agreement between diagnostic tests and Cohen’s kappa (*κ*) statistics with *p* values to measure agreement between diagnostic tests were calculated with the irr package version 0.84.1 in R. Logistic regression was used to regress the asymptomatic *L. donovani* infection (defined as positive for at least one of the four tests) outcome variable onto potential risk factor variables identified in the literature. Latent class analysis was used to estimate diagnostic accuracy and prevalence [22]. Test results were assumed to be conditionally dependent, with Bayesian prior distributions on sensitivity, specificity, and prevalence set using BetaBuster 1.0 (https://betabuster.software.informer.com/). The analysis was implemented in R Studio version 1.1.456 using the ‘lcaR’ model written by Jonathan Marshall (version 2bc8ca6, 13th November 2015) [23].

## Results

### Study population

A total of 720 individuals were sampled, with a median age of 27 years (IQR = 25 years), of whom 280 (38.9%) were male (Table 1). The most common occupations were student (34.4%) and housewife (41.9%) (Table 1). A total of 505 (70.1%) contacts lived within the household of an index case, and 215 (29.9%) lived within a neighbouring household (Table 1).

**Table 1:** Index cases were classified as new VL cases, relapsed VL cases, VL treatment failure, or PKDL

	720 contacts *N* (%)	69 asymptomatic *N* (%)
Median age (IQR)	27 (25)	30 (25)
Male	280 (38.9)	31 (44.9)
Occupation		
Students	248 (34.4)	23 (33.3)
Housewives	301.68 (41.9)	26 (37.7)
Lives within the household of an index case	505 (70.1)	50 (72.5)
VL	476 (66.1)	34 (49.3)
New VL case	429/476 (90.1)	34/34 (100.0)
Relapsed VL case	45/476 (9.5)	0
VL treatment failure	2/476 (0.4)	0
Post–kala azar dermal leishmaniasis (PKDL)	244 (33.9)	35 (50.7)
Macular rash	230/244 (95.0)	33/35 (94.3)
Macular and papular rash	4/244 (1.7)	0
Nodular and macular rash	6/244 (2.5)	0
Macular, nodular, and papular rash	2/244 (0.8)	1/35 (2.9)
Rash type unknown	2/244 (0.8)	1/35 (2.9)

Of the 720 contacts, 476 (66.1%) were associated with VL cases, and 244 (33.9%) were associated with PKDL cases. Of the 69 participants positive for at least one test, 34 (49.3%) were VL cases and 35 (50.7%) were PKDL cases

A total of 69 individuals were positive for at least one diagnostic test, with a median age of 30 (IQR = 25) (Table 1). Of those, 31 (44.9%) were male (Table 1). The most common occupations within the 69 individuals were student (33.3%) and housewife (37.7%), and 50 (72.5%) lived within the household of an index case (Table 1). The 69 asymptomatic cases were spread across 59 (21.4%) of the 276 index cases. Of those 59 index cases, the median percentage positivity of the contacts was 33.3% (IQR = 25).

The 720 contacts were associated with VL cases (66.1%)—made up of new VL cases (90.1%), relapsed VL (9.5%), and VL treatment failure (0.4%)—or PKDL cases (33.9%) (Table 1). Of the 242 PKDL index cases with known rash type, 230 (95.0%) presented with macular rash, four (1.7%) with macular and papular rash, six (2.5%) with nodular and macular rash, and two (0.8%) with macular, nodular, and papular rash (Table 1). The 69 asymptomatic cases were associated with new VL cases (49.3%) or PKDL cases (50.7%), with the majority of such PKDL cases presenting with macular rash (94.3%) (Table 1).

### Estimates of asymptomatic infection in contacts of index cases using tests to detect active infection

Of the 720 participants screened, 69 (9.6%) were positive by at least one test. Of the 720 asymptomatic DNA samples screened, seven (1.0%) were positive by kDNA qPCR, with a mean Ct value of 31.9 (range 26.7–33.9). Urine samples were screened with the *Leishmania* antigen ELISA, of which 24 (3.3%) were found to be positive. Samples screened by DAT were considered positive at a titre of ≥ 1:1600. A total of 28 (3.9%) samples were found to be DAT-positive, 11 (39.3%) of which had a titre ≥ 1:12,800. LAMP detected six (0.8%), eight (1.1%), and three (0.4%) asymptomatic infections when DNA was extracted using Qiagen kits, boil and spin, and from DBS, respectively. For the purposes of further analysis, a participant with a positive LAMP result from any one of the three extraction techniques was considered LAMP-positive, of which there were 15 (2.1%).

**Fig. 1:**
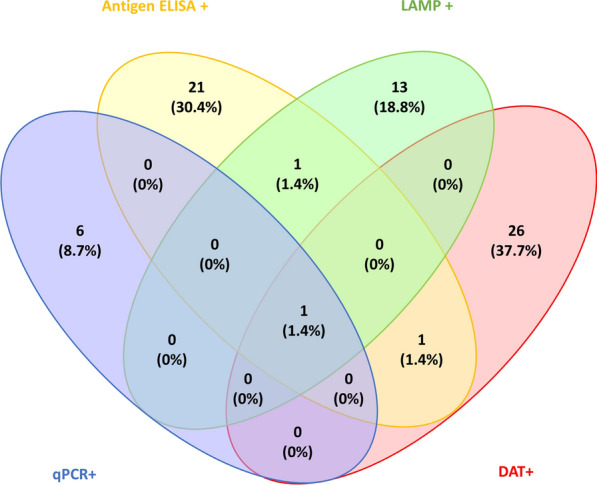
Asymptomatic infection was detected in 69 (9.6%) contacts by a combination of four diagnostic tests. DAT was positive in 28 (40.6%) participants, 26 (37.7%) of whom were positive for DAT alone, and 11/28 (39.3%) of whom had a titre greater than 1:12,800. qPCR was positive in seven (10.1%) participants, six (8.7%) of whom were positive for qPCR alone. LAMP was positive in 15 (21.7%) participants, 13 (18.8%) of whom were positive for LAMP alone. *Leishmania* antigen ELISA was positive in 24 (34.8%) participants, of whom 21 (30.4%) were positive for ELISA alone and three (4.3%) were positive by ELISA and at least one other test

*Leishmania* antigen ELISA and the DAT identified the highest proportion of positive subjects. Only one (0.1%) subject was identified as positive by all four diagnostic methods, two (0.3%) were identified by two diagnostic methods, and 66 (9.2%) were identified by one diagnostic method only. In the 69 asymptomatic participants, 26 (37.7%) were positive by DAT only, and six (8.7%) were positive by qPCR only. Of the 24 (34.8%) participants positive by ELISA, three (4.3%) were positive by at least one other test (Fig. 1). Generally, poor agreement was found between tests. However, antigen and molecular tests showed better agreement in combination compared to the same tests in combination with serology (Table 2). In combination, the DAT and *Leishmania* antigen ELISA capture all participants positive by more than one of the four tests.

**Table 2:** Kappa scores and agreement for four diagnostic tests in 69 asymptomatic participants

Test combination	Agreement (%)	Kappa score	*p* value
DAT and *Leishmania* ELISA	30.4	− 0.476	6.8 × 10^−5^
DAT and qPCR	52.2	− 0.126	0.135
DAT and LAMP	40.6	− 0.330	0.003
*Leishmania* ELISA and qPCR	58.0	− 0.110	0.230
*Leishmania* ELISA and LAMP	49.3	− 0.225	0.049
LAMP and qPCR	71.0	− 0.055	0.614

### Risk factors for asymptomatic VL

Logistic regression was used to confirm risk factors associated with being positive for at least one of the four diagnostic tests. Age, gender, occupation, and living within the index household compared to neighbouring households were not found to be associated with asymptomatic infection. The index case being a PKDL case (OR 1.94, *p* = 0.009), specifically macular PKDL (OR 2.12, *p* = 0.004), was found to be significantly associated with being positive by at least one of the four tests.

### Latent class analysis to estimate infection status and diagnostic accuracy in the absence of a gold standard

In the absence of a single reference standard or a composite reference standard, latent class analysis (LCA) was used to estimate infection status. LCA estimated qPCR, LAMP, DAT, and *Leishmania* antigen ELISA to have sensitivity (2.5–97.5 percentiles) of 85.6% (55.1–99.5), 99.8% (99.2–99.9), 97.5% (90.5–99.9), and 98.9% (96.2–99.9) and specificity of 96.1% (94.7–97.5), 96.7% (95.3–97.8), 99.0% (98.1–99.6), and 97.9% (96.7–98.9), respectively. The prevalence of *L. donovani* asymptomatic infection in VL and PKDL contacts in Bangladesh was estimated to be 0.3% (0.03–0.7).

## Discussion

In this study, we assessed the utility of the DAT, qPCR, LAMP, and *Leishmania* antigen ELISA for detection of asymptomatic *Leishmania* infection in household or neighbouring contacts of VL and PKDL index cases in endemic regions of Bangladesh. Both the DAT and *Leishmania* antigen ELISA capture all samples which are positive by more than one test, and both utilise sample types that have a relatively non-invasive sample collection, which can be transported back to a central laboratory for testing.

The DAT detected the highest proportion of positive individuals. The DAT detects anti-*Leishmania* antibodies that could be circulating from a previously cleared asymptomatic infection. It is not possible to ascertain the time of infection in this cohort as it may be in a symptomatic cohort. However, a recent study found that DAT titres could be a useful tool to monitor transmission in an elimination setting during repeat surveys [14]. Plate-to-plate variation with the DAT in manufacturing and reading, and the relatively low-throughput nature have been previously suggested to be a limitation of this assay for monitoring and surveillance purposes [24]. Our findings are in concordance with previous studies in Bangladesh and India which used serological methods to detect asymptomatic infection [25, 26]. Whereas qPCR requires more laboratory infrastructure, the *Leishmania* antigen ELISA and LAMP are relatively simple techniques suitable for use in resource-poor settings. Furthermore, the *Leishmania* antigen ELISA requires a non-invasive urine sample and is relatively high-throughput, which may aid in screening of high numbers of asymptomatic contacts.

PKDL cases are a potential reservoir of *Leishmania* infection, with experimental infectivity to sand flies estimated to be between 32 and 53% [27]. Here, we demonstrate that a risk factor for asymptomatic infection is living close to a PKDL case, specifically macular PKDL. This follows the launch of the World Health Organization’s road map for neglected tropical diseases 2021–2030, which identifies early detection through methods such as active case detection and development of treatments and diagnostics for both VL and PKDL, as critical actions for the elimination of VL as a public health concern [28]. Our data and the road map highlight the importance of diagnosis and follow-up of PKDL cases, in recognition of their potential role in transmission.

Previous studies have identified risk factors for VL broadly linked to poverty, such as mud walls, with sleeping off the floor found to reduce the risk [29]. Proximity to a previous VL case was identified as a risk factor for VL in Bangladesh [30]. No difference based on sex, occupation, or income, among others, was seen in an analysis of risk factors in the same study [30]. Age trends associated with VL infection were found to vary between studies; however, the prevalence of seropositivity was generally found to increase with age [31].

The specificity of all diagnostics falls below 100% for identification of *L. donovani* asymptomatic infection, according to the LCA conducted, and thus we may expect some false positives on a cohort of this size. This is more probable for the antibody detection test DAT than for the direct detection tests LAMP and antigen ELISA. Therefore, we have looked for overlap in tests which were positive. We acknowledge that sample size may have limited our analysis of risk factors. Additionally, we use latent class analysis to estimate the probability that a participant tests positive at a population level; however, we do not apply this at the individual level for further analysis, given the potential for LCA to be unstable. A further limitation of the study is the lack of follow-up data, and therefore the accuracy of the tests as predictors of progression to clinical disease is unknown.

## Conclusions

In an elimination setting such as Bangladesh, where disease reservoirs are anthroponotic, a relatively simple test such as the *Leishmania* antigen ELISA, which requires a non-invasive urine sample and detects active infection, may be of benefit in combination with serology for surveillance and monitoring of *Leishmania* transmission. Since living with or close to a macular PKDL patient is a risk factor for asymptomatic infection, we propose the follow-up of contacts with PKDL patients as an operational priority. Development of an antigen detection test in RDT format would be of benefit to identify those contacts in the field.

## Data Availability

Data available upon request.

## References

[1] Sengupta PC (1947). History of kala-azar in India. Ind Med Gaz.

[2] Hasker E, Malaviya P, Gidwani K, Picado A, Ostyn B, Kansal S (2014). Strong association between serological status and probability of progression to clinical visceral leishmaniasis in prospective cohort studies in India and Nepal. PLoS Negl Trop Dis.

[3] Singh OP, Hasker E, Sacks D, Boelaert M, Sundar S (2014). Asymptomatic *Leishmania* infection: a new challenge for *Leishmania* control. Clin Infect Dis.

[4] Bern C, Haque R, Chowdhury R, Ali M, Kurkjian KM, Vaz L (2007). The epidemiology of visceral leishmaniasis and asymptomatic leishmanial infection in a highly endemic Bangladeshi village. Am J Trop Med Hyg.

[5] Das VNR, Siddiqui NA, Verma RB, Topno RK, Singh D, Das S (2011). Asymptomatic infection of visceral leishmaniasis in hyperendemic areas of Vaishali district, Bihar, India: a challenge to kala-azar elimination programmes. Trans R Soc Trop Med Hyg.

[6] WHO. Process of validation of elimination of kala-azar as a public health problem in South-East Asia. 2016. https://img1.wsimg.com/blobby/go/c5156b45-48df-4ba4-ab15-be2bb6261d20/downloads/1bu1begqv_714167.pdf.

[7] Muniaraj M (2014). The lost hope of elimination of kala-azar (visceral leishmaniasis) by 2010 and cyclic occurrence of its outbreak in India, blame falls on vector control practices or co-infection with human immunodeficiency virus or therapeutic modalities?. Trop Parasitol.

[8] Ready PD (2014). Epidemiology of visceral leishmaniasis. Clin Epidemiol.

[9] Guarga JL, Lucientes J, Peribáñez MA, Molina R, Gracia MJ, Castillo JA (2000). Experimental infection of *Phlebotomus perniciosus* and determination of the natural infection rates of *Leishmania infantum* in dogs. Acta Trop.

[10] Mondal D, Ghosh P, Chowdhury R, Halleux C, Ruiz-Postigo JA, Alim A (2019). Relationship of serum antileishmanial antibody with development of visceral leishmaniasis, post-kala-azar dermal leishmaniasis and visceral leishmaniasis relapse. Front Microbiol.

[11] Zijlstra EE, Daifalla NS, Kager PA, Khalil EAG, El-Hassan AM, Reed SG (1998). RK39 enzyme-linked immunosorbent assay for diagnosis of *Leishmania donovani* infection. Clin Diagn Lab Immunol.

[12] Chappuis F, Rijal S, Soto A, Menten J, Boelaert M (2006). A meta-analysis of the diagnostic performance of the direct agglutination test and rK39 dipstick for visceral leishmaniasis. BMJ.

[13] Meredith SEO, Kroon NCM, Sondorp E, Seaman J, Goris MGA, Van Ingen CW (1995). Leish-KIT, a stable direct agglutination test based on freeze-dried antigen for serodiagnosis of visceral leishmaniasis. J Clin Microbiol.

[14] Cloots K, Uranw S, Ostyn B, Bhattarai NR, Le Rutte E, Khanal B (2020). Impact of the visceral leishmaniasis elimination initiative on *Leishmania donovani* transmission in Nepal: a 10-year repeat survey. Lancet Glob Health.

[15] Gidwani K, Picado A, Ostyn B, Singh SP, Kumar R, Khanal B (2011). Persistence of *Leishmania donovani* antibodies in past visceral leishmaniasis cases in India. Clin Vaccine Immunol.

[16] Hossain F, Ghosh P, Khan MAA, Duthie MS, Vallur AC, Picone A (2017). Real-time PCR in detection and quantitation of *Leishmania donovani* for the diagnosis of visceral leishmaniasis patients and the monitoring of their response to treatment. PLoS ONE.

[17] Adams ER, Schoone G, Versteeg I, Gomez MA, Diro E, Mori Y (2018). Development and evaluation of a novel loop-mediated isothermal amplification assay for diagnosis of cutaneous and visceral leishmaniasis. J Clin Microbiol.

[18] Mukhtar M, Ali SS, Boshara SA, Albertini A, Monnerat S, Bessell P (2018). Sensitive and less invasive confirmatory diagnosis of visceral leishmaniasis in Sudan using loop-mediated isothermal amplification (LAMP). PLoS Negl Trop Dis.

[19] Vallur AC, Tutterrow YL, Mohamath R, Pattabhi S, Hailu A, Abdoun AO (2015). Development and comparative evaluation of two antigen detection tests for visceral leishmaniasis. BMC Infect Dis.

[20] Hasker E, Kansal S, Malaviya P, Gidwani K, Picado A, Singh RP (2013). Latent Infection with *Leishmania donovani* in highly endemic villages in Bihar, India. PLoS Negl Trop Dis.

[21] Oliveros JC. Venny. An interactive tool for comparing lists with Venn’s diagrams. (2007–2015). https://bioinfogp.cnb.csic.es/tools/venny/index.html.

[22] van Smeden M, Naaktgeboren CA, Reitsma JB, Moons KGM, de Groot JAH (2014). Latent class models in diagnostic studies when there is no reference standard–a systematic review. Am J Epidemiol.

[23] Marshall J. Latent class analysis in R. 2015. https://raw.githubusercontent.com/jmarshallnz/lcar/master/lca.R.

[24] Vallur AC, Reinhart C, Mohamath R, Goto Y, Ghosh P, Mondal D (2016). Accurate serodetection of asymptomatic *Leishmania donovani* infection by use of defined antigens. J Clin Microbiol.

[25] Banu SS, Meyer W, Ahmed B-N, Kim R, Lee R (2016). Detection of *Leishmania donovani* in peripheral blood of asymptomatic individuals in contact with patients with visceral leishmaniasis. Trans R Soc Trop Med Hyg.

[26] Rabi Das VN, Bimal S, Siddiqui NA, Kumar A, Pandey K, Sinha SK (2020). Conversion of asymptomatic infection to symptomatic visceral leishmaniasis: a study of possible immunological markers. PLoS Negl Trop Dis.

[27] Hirve S, Boelaert M, Matlashewski G, Mondal D, Arana B, Kroeger A (2016). Transmission dynamics of visceral leishmaniasis in the Indian Subcontinent—a systematic literature review. PLoS Negl Trop Dis.

[28] World Health Organisation. Ending the neglect to attain the sustainable development goals: a road map for neglected tropical diseases 2021–2030. 2020. http://apps.who.int/bookorders.

[29] Bern C, Courtenay O, Alvar J (2010). Of cattle, sand flies and men: a systematic review of risk factor analyses for South Asian visceral leishmaniasis and implications for elimination. PLoS Negl Trop Dis.

[30] Bern C, Hightower AW, Chowdhury R, Ali M, Amann J, Wagatsuma Y (2005). Risk factors for kala-azar in Bangladesh. Emerg Infect Dis.

[31] Chapman LAC, Morgan ALK, Adams ER, Bern C, Medley GF, Dé Irdre Hollingsworth T (2018). Age trends in asymptomatic and symptomatic *Leishmania donovani* infection in the Indian subcontinent: a review and analysis of data from diagnostic and epidemiological studies. PLoS Negl Trop Dis.

